# Digitale Maßnahmen der Kundenkommunikation im inhabergeführten Einzelhandel zwischen kurzfristiger Reaktion auf die Coronapandemie und zukunftsfähiger Strategie

**DOI:** 10.1007/s00548-023-00869-8

**Published:** 2023-06-13

**Authors:** Elisa Gliesner, Daniel Schiller

**Affiliations:** 1 Deutsche Stadt- und Grundstücksentwicklungsgesellschaft mbH, Leipzig, Deutschland; 2 Institut für Geographie und Geologie, Universität Greifswald, Greifswald, Deutschland

**Keywords:** Digitalisierung, Einzelhandel, Online-Marktplatz, Multi-channel shop, New social commerce, Digitalisation, Retail trade, Online marketplace, Multi-channel shop, New social commerce

## Abstract

Der inhabergeführte Einzelhandel steht vor besonderen Herausforderungen bei der Einführung und Etablierung digitaler Kundenkommunikationsmaßnahmen (KKM). Im vorliegenden Beitrag wird untersucht, welche Maßnahmen im Zuge der Coronapandemie eingeführt worden sind und welche dieser Maßnahmen besonders zukunftsfähig sein können. Darüber hinaus wird die Bedeutung von regionalen Netzwerken beim Wissenstransfer über digitale Maßnahmen betrachtet. Für die Untersuchung wurde in den drei Bundesländern Nordrhein-Westfalen, Sachsen und Sachsen-Anhalt im März 2022 eine Online-Befragung von Inhabern durchgeführt und durch fünf Experteninterviews im April und Mai 2022 ergänzt. Für die Vergleichbarkeit der gewonnenen Ergebnisse wird eine großflächige Handelsstudie der IHK Köln und dem ibi Research Institut Regensburg aus dem Jahr 2020 herangezogen. Dabei stellte sich heraus, dass insbesondere die digitale Sichtbarkeit für den Erhalt der inhabergeführten Einzelhandelsgeschäfte in Zukunft von zentraler Bedeutung sein wird. Ferner präsentieren sich die sozialen Medien mit ihrer perspektivisch ausgestatteten Verkaufsfunktion als geeignetes Instrument. Außerdem konnten durch eine differenzierte Betrachtung von Branchen und Zielgruppen sowie unterschiedlichen Ansätzen für den Einstieg in die digitale Kundenkommunikation zukunftsfähige und individuelle Maßnahmen identifiziert werden.

## Abstract (en)

Owner-managed retail faces special challenges when introducing and establishing digital customer communication measures. This article examines which measures have been introduced in the course of the COVID-19 pandemic and which of these measures may be particularly sustainable. In addition, the importance of regional networks in knowledge transfer via digital measures is considered. For the study, an online survey of owners was carried out in the three federal states of North Rhine-Westphalia, Saxony and Saxony-Anhalt in March 2022 and was supplemented by five expert interviews in April and May 2022. A large-scale trade study by the Cologne Chamber of Industry and Commerce and the ibi Research Institute Regensburg from 2020 was used to compare the results obtained. As a result of the study, digital visibility was found to be of particular importance for the preservation of owner-managed retail stores in the future. Furthermore, social media are represented as a suitable instrument with their prospectively provided selling function. In addition, sustainable and individual measures could be identified through differentiated consideration of sectors and target groups as well as different approaches to entering digital customer communication.

## Einleitung

**Abb. 1:**
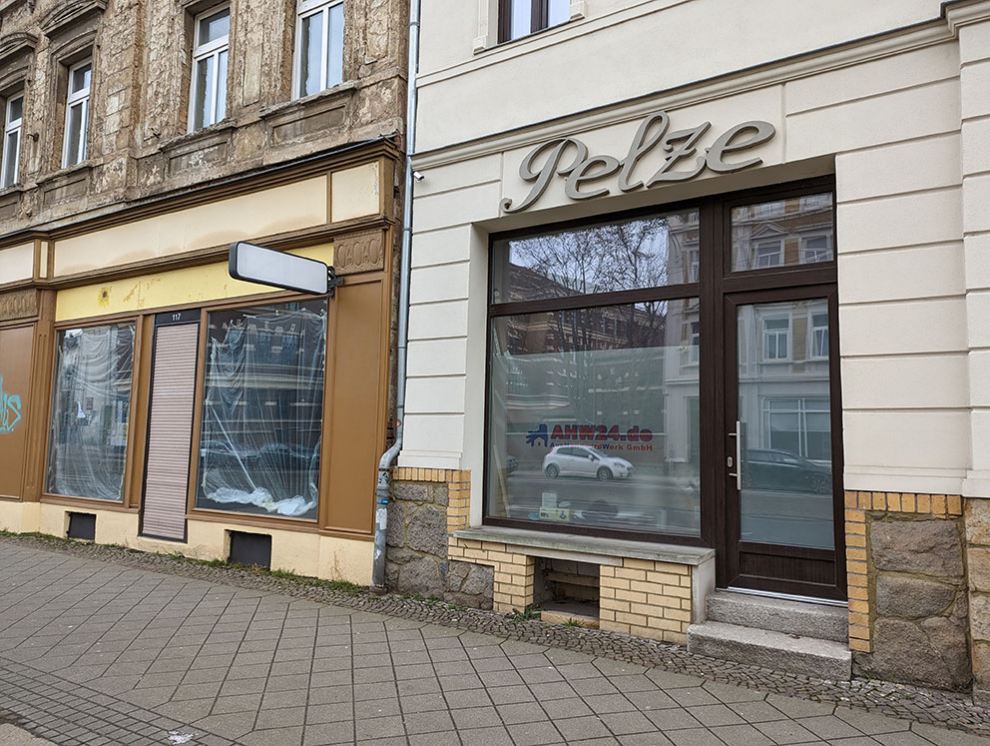
Der inhabergeführte Einzelhandel steht unter verstärktem Anpassungsdruck. (Aufnahme: Autor, Leipzig)

„Stillstand in der Branche ist tödlich. Man muss sich der Gesellschaft anpassen – und die ist seit zwei Jahren gezwungen, ihr Einkaufsverhalten zu ändern“, schreibt ein Inhaber in der Online-Befragung und bezieht sich damit auf die aktuellen Entwicklungen der Digitalisierung im inhabergeführten Einzelhandel. Dieser wuchs in den vergangenen Jahren stetig zwischen 9 und 12 %. Mit der Coronapandemie stieg das Wachstum 2020 auf 23 % an und liegt 2021 bei 19 % (HDE 2022, S. 3–7). Auch das veränderte Kaufverhalten der Kunden begünstigt den Onlinehandel in den vergangenen Jahren, wobei weiterhin der Großteil des Umsatzes im stationären Handel erfolgt. Im Rahmen einer Studie der ibi Research (Paul und Stahl 2020, S. 8) gaben 51 % von 1007 befragten Kunden an, dass sie bereits am liebsten bzw. alles im Internet einkaufen. Nur 10 % kaufen ausschließlich oder bevorzugt im stationären Geschäft ein. Erschwerend kommt für den stationären Einzelhandel hinzu, dass sich viele Innenstädte mit Verödungen oder Vereinheitlichung durch Leerstände (vgl. Abb. 1) bzw. Filialisierung konfrontiert sehen (Gruninger-Hermann 2017). Infolgedessen ist beispielsweise durch steigende Mieten in den „1a-Lagen“ oder einer Nachfolgeproblematik hauptsächlich der inhabergeführte Einzelhandel gezwungen, in unattraktivere Nebenlagen zu ziehen oder das Geschäft aufzugeben (Schade et al. 2021). Das „Handelsszenario 2030“ der IFH Köln prognostiziert, dass in den kommenden zehn Jahren 24.000–64.000 Unternehmen schließen müssen (REMIRA Group GmbH 2022). Es stellt sich nunmehr die Frage, wie der stationäre Einzelhandel angesichts der immer größer werdenden Konkurrenz des Onlinehandels und anderen Herausforderungen weiterhin am Wettbewerb teilhaben kann.

Als Antwort auf diese Frage und auch im Zuge der Lockdowns während der Coronapandemie haben viele Einzelhändler digitale Kundenkommunikationsmaßnahmen (KKM) eingeführt bzw. genutzt (HDE 2021). In vorangegangenen Studien wie die der IHK-ibi (Wittmann und Deichner 2020) wurden stets viele verschiedene Händlergruppen zu ihrer Nutzung von digitalen KKM befragt, aber keine klare Unterscheidung des Betriebstyps in der Ergebnispräsentation vorgenommen. Aus diesem Grund wird der Fokus der Forschung auf den inhabergeführten Einzelhandel und seinen Umgang bzw. Fortschritt in der Digitalisierung der Kundenkommunikation gelegt. Darüber hinaus identifizierte die bisherige Forschung insbesondere für diesen einen Nachholbedarf in der Digitalisierung (Heinemann 2021).

Der Beitrag untersucht daher, welche der eingeführten digitalen KKM für den inhabergeführten Einzelhandel zukunftsfähig sein können oder lediglich eine kurzfristige Reaktion auf die Lockdowns dargestellt haben. Des Weiteren beschäftigt er sich mit der Rolle von lokalen bzw. regionalen Netzwerken und ihrem Beitrag zu Wissenstransfers über digitale Maßnahmen und deren Umsetzung. Ziel des Beitrags ist es, zukunftsfähige sowie erfolgreiche digitale KKM herauszuarbeiten und Handlungsempfehlungen für den inhabergeführten Einzelhandel auszusprechen.

## Digitalisierung im Einzelhandel

Der Online-Handel zählt zum Distanzhandel. Dieser beschreibt einen interaktiven Handel, bei dem Verkäufer und Käufer nicht in physischen Kontakt treten und somit durch eine räumliche Trennung gekennzeichnet sind. Demgegenüber steht der Residenzhandel, der den klassischen stationären Handel beschreibt. Durch die Nutzung des Internets hat sich eine neue Mischform herausgebildet, die als „Hybridprinzip“ bezeichnet wird. Die Internetnutzung dient dabei zur Vorbereitung oder Unterstützung des Kaufs im stationären Geschäft bzw. andersherum (Heinemann 2021).

**Abb. 2:**
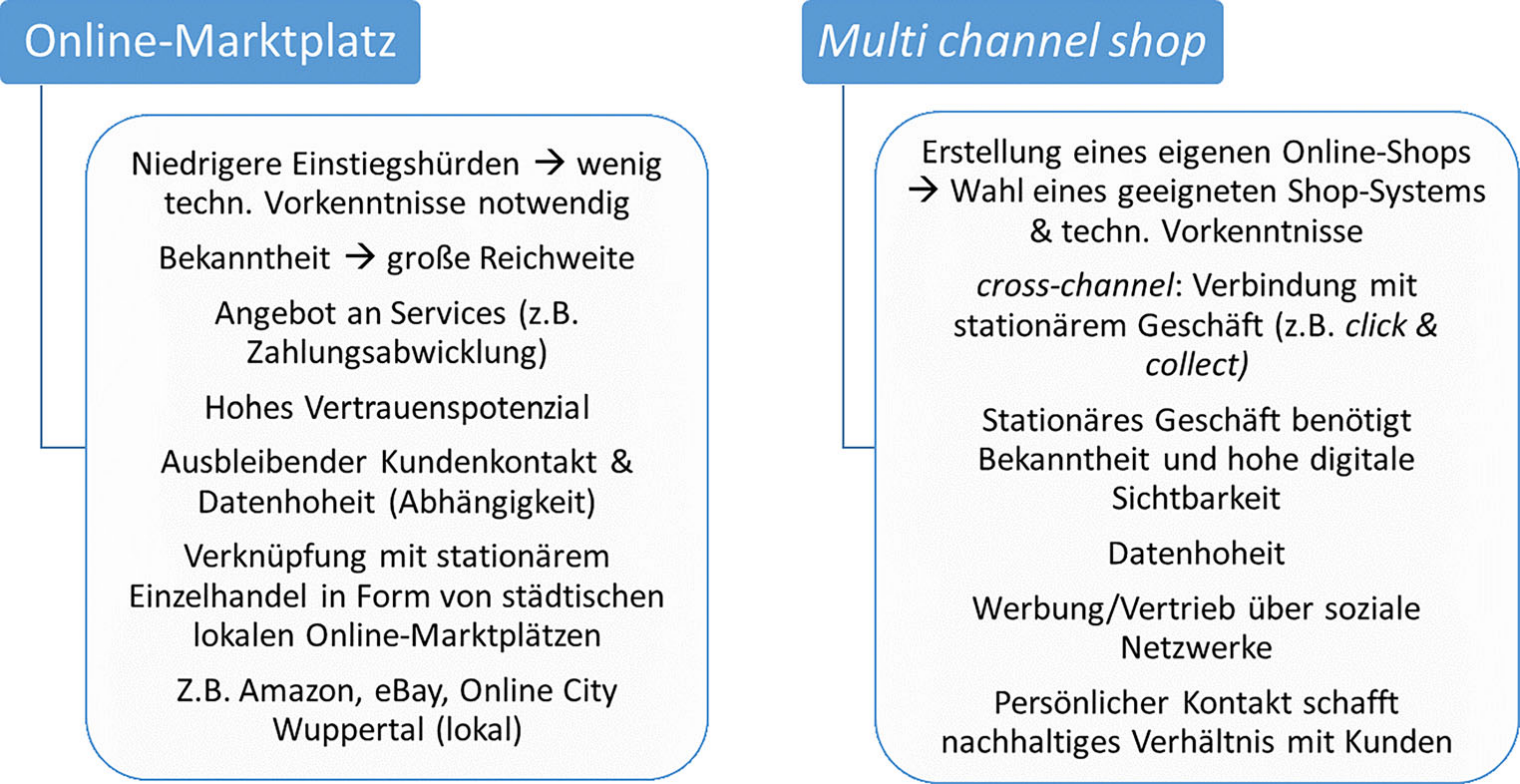
Vor- und Nachteile der Betriebstypen im Vergleich. (Eigene Darstellung)

Von den verschiedenen Betriebstypen des Onlinehandels sind für diesen Beitrag der Online-Marktplatz (OM) sowie der *multi channel shop* von Interesse, da diese mit einem stationären Geschäft verknüpft werden können. Abb. 2 zeigt einige Vor- und Nachteile des jeweiligen Betriebstyps für den inhabergeführten Einzelhandel auf. Für den Einstieg in jeglichen Onlinevertrieb ist das Vorhandensein eines digitalen Warenwirtschaftssystems zwingend notwendig. Bei den OM wird ein besonderes Augenmerk auf die lokalen Online-Marktplätze (LOM) gelegt. Diese sollen den stationären Einzelhandel in einer abgegrenzten Region unterstützen, indem sie lokale Händler in den Vordergrund stellen und online sichtbar machen (Neiberger 2020). Dadurch können Wertschöpfungsketten regional reorganisiert und durch professionelle Abläufe im LOM Wettbewerbsvorteile generiert werden, die sonst nur den „Big Playern“ vorbehalten sind (Hardaker 2022).

Digitale KKM haben das große Potenzial, über die bloße digitale Sichtbarkeit eines Google-My-Business-Eintrags oder einer Homepage hinauszugehen. An dieser Stelle setzen insbesondere *multi channel shops* an, die auch eine Nutzung sozialer Medien und *new social commerce *umfassen. Der Vorteil besteht in dem direkten Austausch mit den Kunden in Form von Chat-Nachrichten, Live-Shopping-Formate oder anhand von Kommentaren und Likes. Darüber hinaus werden soziale Medien von Unternehmen für die Kundenbindung sowie -betreuung genutzt (Heinemann 2021). Die Bedeutung der sozialen Netzwerke nimmt außerdem stetig zu, sodass von einem Verlust von Wettbewerbsvorteilen infolge ihrer Nichtverwendung gesprochen wird (Corduan 2018). Ein weiterführender Schritt von der alleinigen Kommunikation und Werbung ist der Weg hin zum *new social commerce*. In diesem Fall wird zusätzlich eine Transaktionsmöglichkeit angeboten. Das bedeutet, dass dem Kunden ermöglicht wird, Waren oder Dienstleistungen auf sozialen Medien käuflich zu erwerben. Diese direkte Verkaufsfunktion auf der Plattform kann vor allem kleineren Händlern ohne Online-Shop die Möglichkeit bieten, ihre Produkte online zu vertreiben. Außerdem werden Hürden für den Kauf verringert, da für die Kunden der Weg in den Online-Shop, auf die Website oder das persönliche Kontaktieren der Anbieter entfallen (Heinemann 2021).

Bei der Betrachtung der Affinität zur Digitalisierung des deutschen Einzelhandels zeigen sich deutliche Unterschiede zwischen den einzelnen Organisationsformen. Filialisten und Franchise-Unternehmen verfügen in der Regel über einen Online-Shop, mindestens aber über eine Homepage. Der inhabergeführte Handel hingegen ist bisher zu einem großen Teil noch gar nicht digitalisiert (Neiberger und Kubon 2018), wodurch ein Nachholbedarf bei der Digitalisierung attestiert wird (Heinemann 2021). In dem von Friedrich et al. (2022) gewählten Untersuchungsgebiet einer Vergleichsstudie hat sich trotz des gestiegenen Handlungsdrucks durch die Coronapandemie der Anteil der Unternehmen, die keinen Online-Auftritt besitzen, zwischen 2017 und 2021 nur von 45 % auf 41 % reduziert. Die Nutzung von sozialen Medien ist allerdings um 23 % angestiegen. Als Gründe für die ausbleibende Digitalisierung werden hauptsächlich fehlende Ressourcen genannt. Darunter fällt der personelle, finanzielle sowie zeitliche Aspekt und das fehlende Know-how.

## Methodisches Vorgehen

Für diesen Beitrag wurden Daten zu digitalen Maßnahmen der Kundenkommunikation im inhabergeführten Einzelhandel und zu ihrer Zukunftsfähigkeit durch eine standardisierte Befragung im März 2022 und ergänzende qualitative Interviews im April und Mai 2022 generiert. Zum einen wurde in drei Bundesländern (Nordrhein-Westfalen, Sachsen und Sachsen-Anhalt) eine Online-Befragung von Inhabern stationärer Einzelhandelsgeschäfte mithilfe eines standardisierten Fragebogens durchgeführt. Durch die Unterschiedlichkeit der drei Bundesländer hinsichtlich ihrer Wirtschaftskraft, aber auch der Bevölkerungsdichte und der ländlichen bzw. städtischen Prägung wird der inhabergeführte Einzelhandel in Deutschland damit in seiner Vielfalt abgebildet. Insgesamt konnten von 957 selbst online recherchierten und kontaktierten Inhabern aus 43 Städten und einer Streuung der Umfrage über verschiedene Verbände 182 Inhaber (gültige Fälle) von stationären Einzelhandelsgeschäften befragt werden. Dabei nahmen 131 in Nordrhein-Westfalen, 24 in Sachsen und 27 Inhaber in Sachsen-Anhalt an der Online-Befragung teil. In der Befragung wurden die Warengruppe, die Nutzung verschiedener digitaler KKM (in Anlehnung an die ibi Research-Befragung) zu verschiedenen Zeitpunkten (vor/während/nach der Corona-Pandemie), die Informationsquellen, die Einschätzung des Wissens über die Digitalisierung sowie der Umsatz und die Effekte der Digitalisierung abgefragt. Die Ergebnisse der Befragung wurden in Microsoft Excel u. a. mithilfe von Pivot-Tabellen ausgewertet und grafisch dargestellt.

Zum anderen wurden fünf problemzentrierte, leitfadengestützte Experteninterviews mit zwei Citymanagern, einem Wirtschaftsförderer mit Verbindung zur Online-City Wuppertal und je einem Referenten des Stadtmarketings sowie digitalen Handels durchgeführt. Für die Experteninterviews sollten Interviewpartner akquiriert werden, die sich primär mit der Digitalisierung des Einzelhandels beschäftigen und gleichzeitig in einem Netzwerk agieren. Als Netzwerk kamen Verbände, Initiativen, Institutionen oder städtische Einrichtungen wie die Wirtschaftsförderung, das Citymanagement oder das Stadtmarketing infrage. Eine örtliche Begrenzung auf die zuvor definierten Untersuchungsgebiete war nicht erforderlich, da vielmehr eine zusätzliche, bundesweite Gesamteinschätzung durch die Experteninterviews erwünscht ist. In verschiedenen Quellen wurde der lokale Online-Marktplatz „Online City Wuppertal“ aufgegriffen, bei dem es sich um ein Pilotprojekt handelt. Die Erfahrungen aus diesem Projekt sind dabei von Interesse für diesen Artikel. Der Leitfaden wurde in vier Kategorien geteilt: 1) Angaben zum Interviewpartner: Vorstellung, Angaben zum Tätigkeitsbereich und zum Vorhandensein von digitalen Angeboten; 2) Digitale KKM: Bedeutung für den inhabergeführten Einzelhandel, Gründe für die geringe Digitalisierung, am häufigsten genutzte Maßnahmen, Abgabe einer Empfehlung; 3) Rolle von Netzwerken: Informationsquellen, Wissenstransfers, Akzeptanz der Netzwerke, Verbesserungsmöglichkeiten; 4) Abschlussfrage: Einschätzung, wo sich der inhabergeführte Einzelhandel in zehn Jahren mit/ohne Digitalisierung befindet. Die Interviews wurden zur weiteren Verwendung transkribiert sowie kodiert.

## Digitale Kundenkommunikation in den Untersuchungsregionen

### Nutzung digitaler Maßnahmen zur Kundenkommunikation

**Abb. 3:**
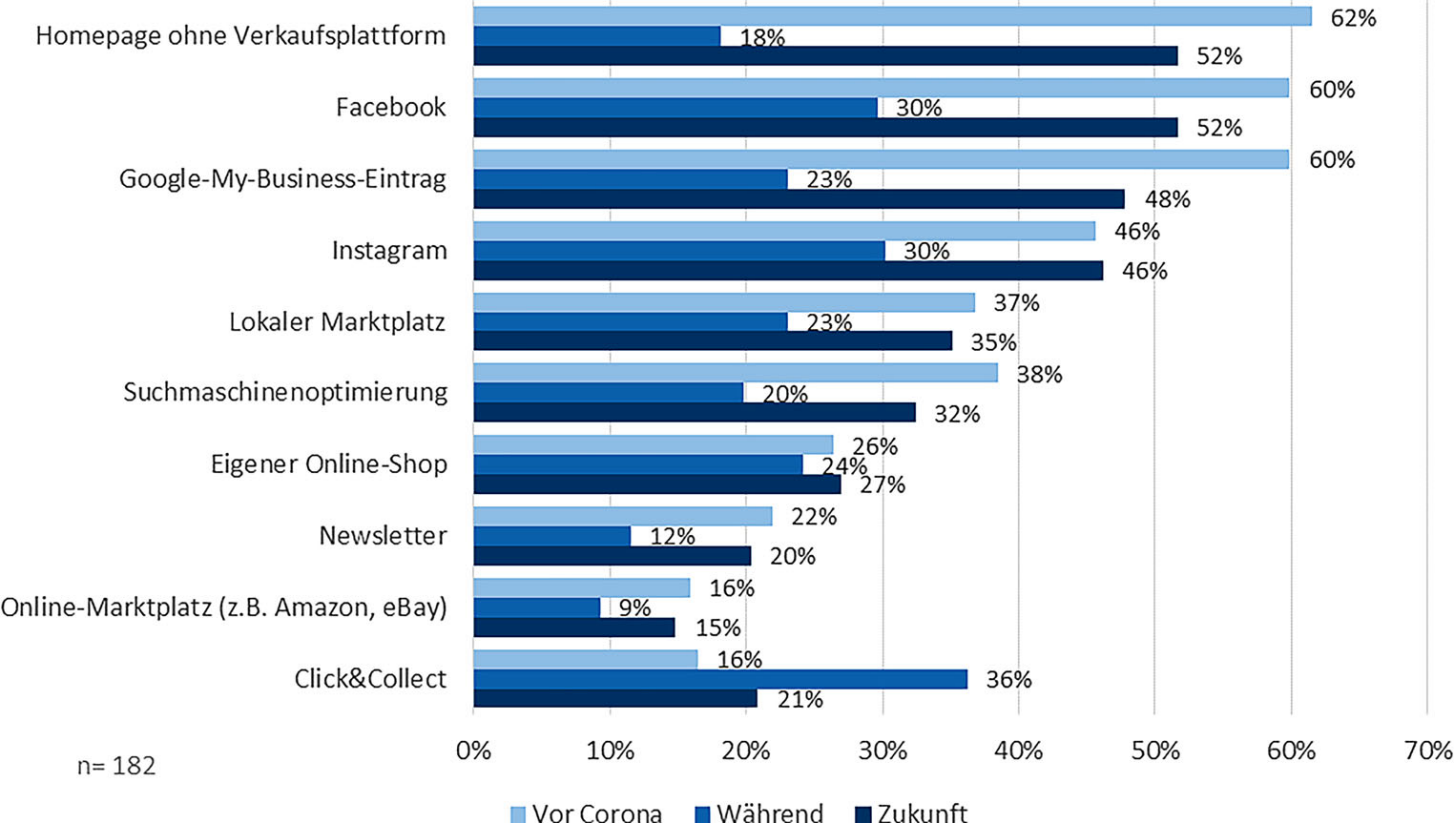
Nutzung von digitalen KKM zu verschiedenen Zeitpunkten. (Eigene Darstellung)

Alle interviewten Experten sind sich einig, dass sowohl die digitale Kundenkommunikation als auch die Partizipation in einem Netzwerk für den inhabergeführten Einzelhandel sehr wichtig und notwendig sind. Abb. 3 (in Anlehnung an die zweite IHK-ibi-Handelsstudie) zeigt, dass die Homepage, die sozialen Medien und der Google-My-Business-Eintrag am häufigsten sowohl vor als auch nach der Pandemie genutzt werden. Besonders auffällig ist der starke Zuwachs der Nutzung von *click&collect *während der Pandemie auf über das Doppelte gegenüber der Zeit davor. Des Weiteren zeigt sich im Vergleich der OM mit den LOM, dass lokale Angebote grundsätzlich häufiger genutzt werden als die „Big Player“. An dieser Stelle ist darauf hinzuweisen, dass die teilweise erzwungene vorübergehende Schließung von Geschäften während der Coronapandemie scheinbar dazu geführt hat, dass viele Händler auch ihre Online-Präsenzen nicht weitergepflegt haben. Dies könnte erklären, warum nur ein geringer Anteil der Befragten in der Zeit der unmittelbaren Pandemie seine digitalen KKM auf- bzw. ausgebaut hat. Lediglich *click&collect* konnte während der Pandemie einen deutlichen Nutzungszuwachs erzielen. Eine umfassende weiterführende Verwendung lässt sich an den Ergebnissen allerdings nicht ablesen, wodurch von einer kurzfristigen, meist auch weniger professionellen umgesetzten Reaktion gesprochen werden kann. Auch generell ist zu beobachten, dass die Anteile der geplanten Nutzung in der Zeit nach der Pandemie sich in vielen Bereichen nicht wesentlich von denen vor der Pandemie unterscheiden. Ein breit angelegter Digitalisierungsschub im inhabergeführten Einzelhandel lässt sich aus diesen Ergebnissen daher nicht ableiten.

**Abb. 4:**
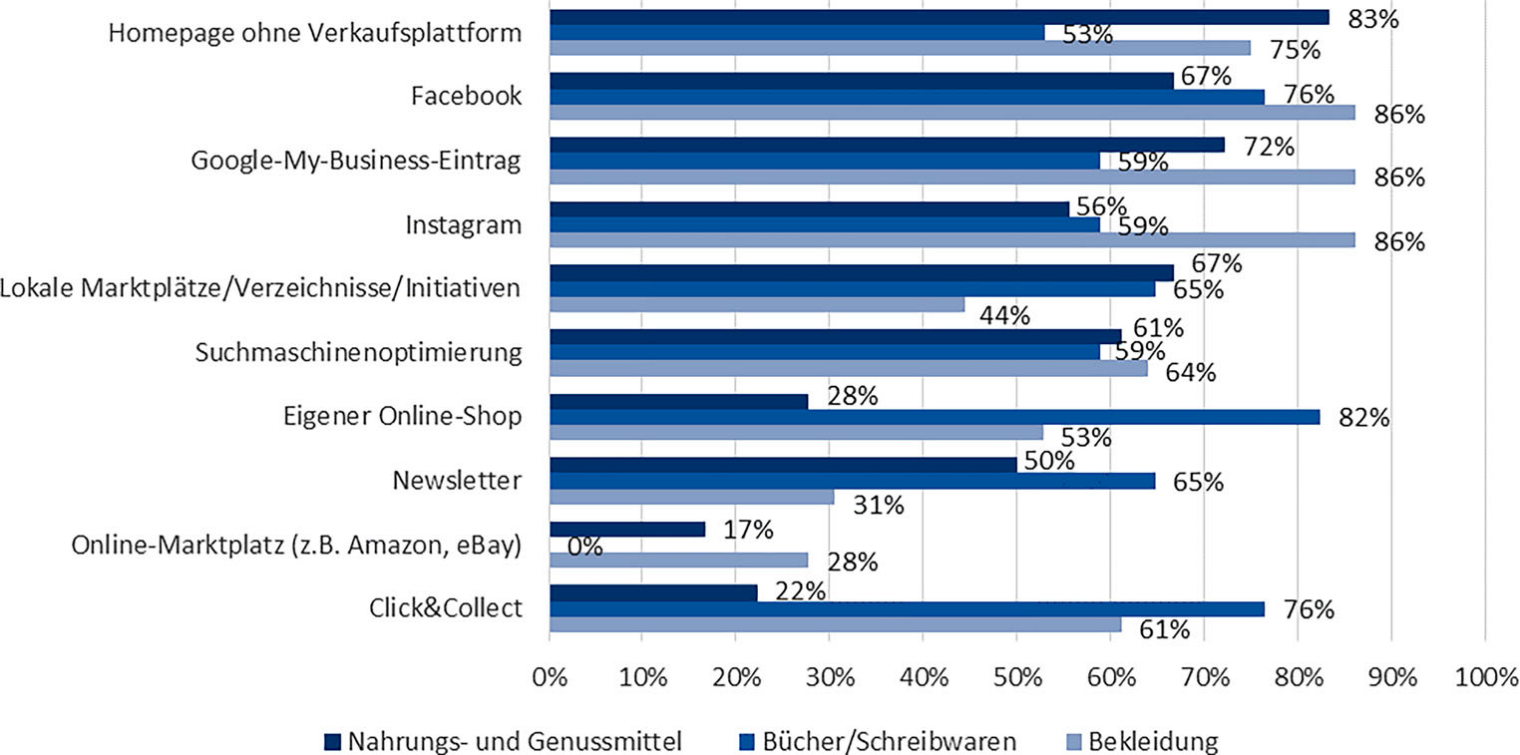
Nutzung von digitalen KKM im Branchenvergleich. (Eigene Darstellung)

Im Branchenvergleich (vgl. Abb. 4) zeigen sich klare Unterschiede in der Nutzung der digitalen KKM. Während die präsentationsintensivere Bekleidungsbranche die sozialen Medien bevorzugt, sind es bei der Bücher- und Schreibwarenbranche der eigene Online-Shop in Verbindung mit *click&collect*. Die Branche der Nahrungs- und Genussmittel, die eher kurzfristige Bedarfe deckt, nutzt am häufigsten die Homepage und zeigt im Vergleich zu den anderen beiden Branchen eine Unterrepräsentation eigener Online-Shops und *click&collect*.

### Empfehlungen zum Ausbau der Nutzung

**Abb. 5:**
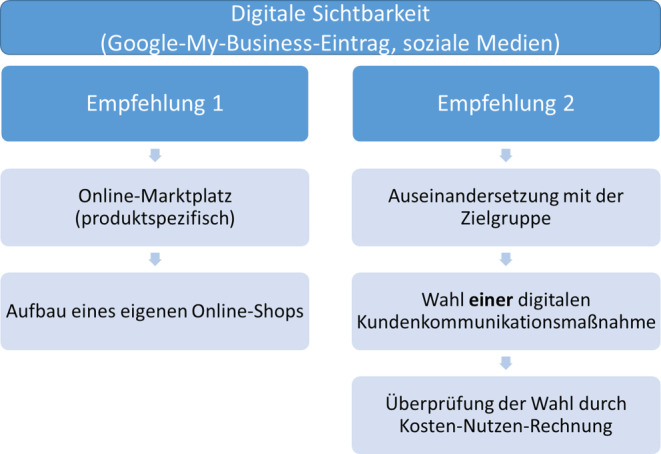
Empfehlung zur Nutzung von digitalen KKM zum Einstieg. (Eigene Darstellung)

Im Rahmen der Interviews wurde nach Empfehlungen gefragt, wie ein Einstieg in die digitale Kundenkommunikation erfolgreich gemeistert werden kann. Aus den Antworten konnten zwei Empfehlungsbündel abgeleitet werden, die in Abb. 5 grafisch dargestellt werden.

Bei der Betrachtung der beiden Empfehlungen der Experten ist festzustellen, dass diese teilweise im Widerspruch stehen und insbesondere die erste Empfehlung durch die Online-Befragung der Händler hinsichtlich ihrer breiten Akzeptanz kritisch zu diskutieren ist. Einer Etablierung von Online-Shops steht ein unverhältnismäßig hoher personeller und zeitlicher Aufwand bei der Implementierung und Pflege entgegen. Entsprechend der Befragungsergebnisse und anderer Untersuchungen präsentiert sich daher die Empfehlung 2 für online unerfahrene Inhaber als ratsamer, da in diesem Kontext sowohl die vorhandenen zeitlichen und personellen Ressourcen als auch die Branche und ihre jeweilige Zielgruppe betrachtet und berücksichtigt werden, um anschließend eine passende digitale KKM auszuwählen.

### Bedeutung von Netzwerken

Bei der Befragung der Inhaber stellte sich heraus, dass 61 % in mindestens einem Netzwerk in Form einer Mitgliedschaft oder Partizipation aktiv sind. Die Netzwerke werden als sehr hilfreich bei der Umsetzung von Digitalisierungsmaßnahmen wahrgenommen und haben durch die Coronapandemie nach Aussage der Interviewpartner weiter an Mitgliedern gewonnen. Hauptsächlich werden dabei Vorträge, Workshops und persönliche Beratungen angeboten, aber auch das Publizieren und Weiterleiten von Informationen ist Netzwerkaufgabe. Die Angebotsdarreichung erfolgt dabei niedrigschwellig. Verbesserungsmöglichkeiten werden zum einen in der regionalen Aufklärungsarbeit vor Ort gesehen, zum anderen in der Verstetigung und Förderung von Netzwerkarbeit in Innenstädten. Grundsätzlich fasst es ein Experte wie folgt zusammen: „Ohne Netzwerk funktioniert inhabergeführter Einzelhandel nicht“, da durch diese wichtige Synergieeffekte und Informationsaustausch stattfinden. Dies schließt an dieser Stelle auch den Austausch in informellen Netzwerken, wie bspw. in einer Händlergemeinschaft, ein.

## Diskussion

Aus der standardisierten Befragung und den Experteninterviews hat sich vor allem die Verwendung von sozialen Medien als für die Zukunft empfehlenswert herausgestellt. Sie werden nicht nur von fast allen Bevölkerungsgruppen genutzt, sondern tragen als digitales Schaufenster zu einer besseren Wahrnehmung des Geschäfts und der Produkte bei. Durch den persönlichen Kontakt wird zudem die Kundenbindung gestärkt. Mit der Einführung von *new social commerce* wird der direkte in-App-Kauf von Produkten über die sozialen Medien ermöglicht und auf die Weiterleitung auf eine Verkaufswebsite verzichtet. Dabei stellt sich die Frage, inwieweit diese Funktion von den Kunden angenommen wird und ob seine Einführung die Nutzung eines Online-Shops durch schnelles Einkaufen direkt am Smartphone auf lange Sicht ersetzen kann.

**Abb. 6:**
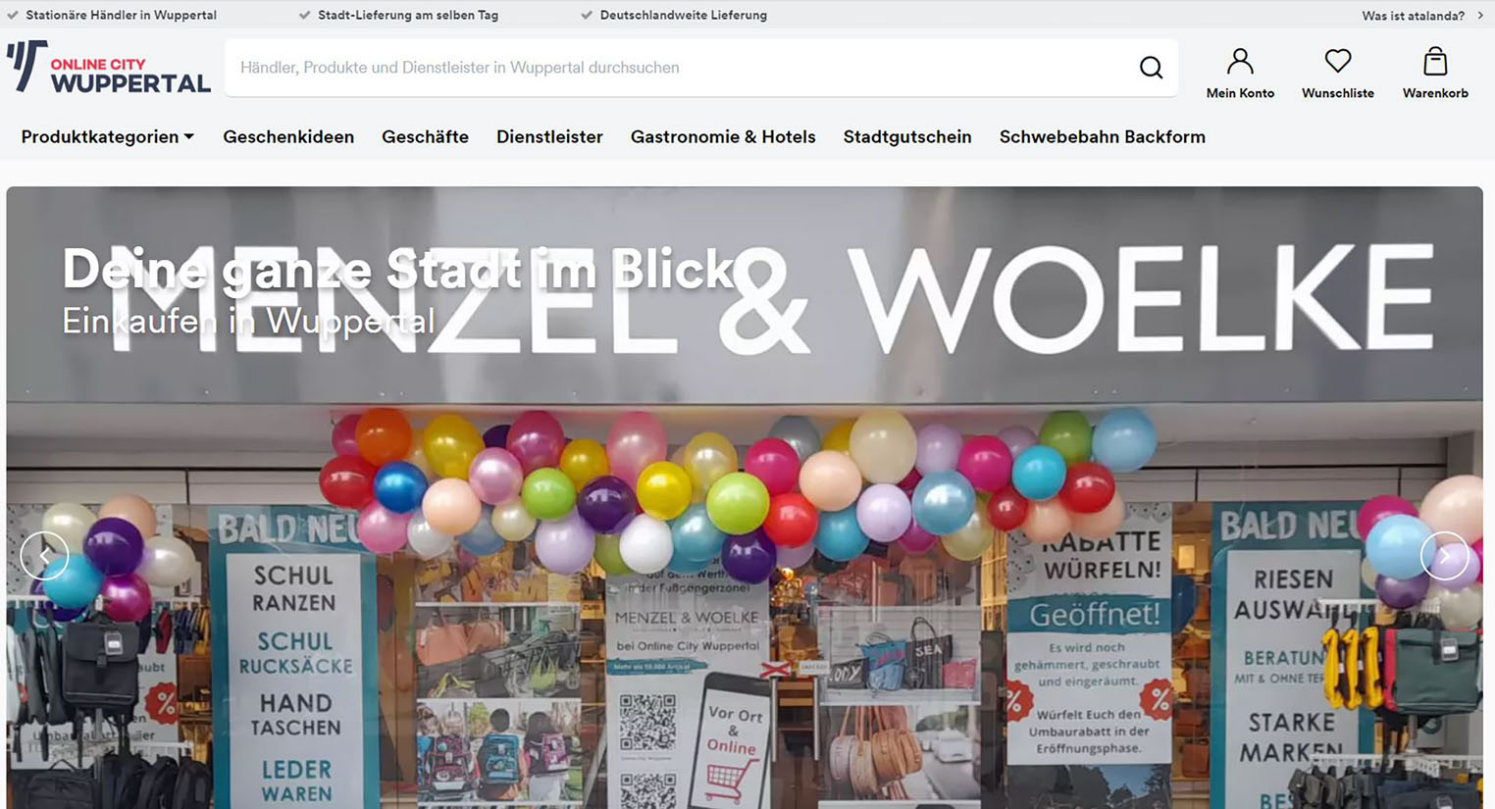
Online City Wuppertal als Beispiel für einen lokalen Online-Marktplatz. (Quelle: https://atalanda.com/wuppertal)

Beachtenswert ist ebenfalls die Nutzung von OM und LOM. OM werden auf Basis dieser Untersuchung von den Inhabern in allen Branchen am wenigsten bis gar nicht genutzt, während die LOM bereits eine gewisse Beliebtheit besitzen (vgl. Abb. 6). Auf diesen wird eine persönliche Ebene geschaffen und somit die Kundenbindung im regionalen Kontext, aber auch überregional gestärkt. Durch die ebenfalls professionelle Betreuung der Plattform wird ein Service für die Kunden kreiert und für Werbemaßnahmen gesorgt. Im Idealfall können viele Vorteile der großen OM auch im lokalen Kontext generiert werden, aber ohne die Anonymität und die großen Machtasymmetrien zwischen Betreibern der OM und den Händlern. LOM bieten damit für den inhabergeführten Einzelhandel eine besonders attraktive (Verkaufs‑)Plattform, welche auch auf Homepages oder sozialen Medien verlinkt werden kann. LOM bieten auch eine sehr gute Verbindung zu den ebenfalls als wirksam herausgearbeiteten Netzwerkstrukturen vor Ort. Damit ist die Etablierung von LOM eine Aufgabe, die beispielsweise von Wirtschaftsförderung oder Citymanagement aufgegriffen werden kann. Die dafür notwendige Etablierung digitaler Warenwirtschaftssysteme durch die Händler kann allerdings für digital Unerfahrene als eine Hürde identifiziert werden, um eine große Zahl an regionalen Anbietern für einen LOM zu gewinnen. Eine Lösungsmöglichkeit könnte darin bestehen, weiterführende Beratungsleistungen zu notwendigen Voraussetzungen und den richtigen digitalen Auftritt (Produktbeschreibung und -darstellung) zu organisieren. Dennoch ist anzumerken, dass laut Medienberichten zufolge LOM teilweise als „gescheitert“ erklärt werden (Pohlgeers 2019) und es fraglich ist, wie sich diese nach der Pandemie entwickeln und ob sie weiterhin Zuspruch bei den Inhabern finden.

## Fazit

Die Untersuchung zeigt, dass insbesondere die digitale Sichtbarkeit für den inhabergeführten Einzelhandel von essenzieller Bedeutung ist und die Informationen aktuell und stetig gepflegt werden sollten. Die digitale Sichtbarkeit kann auf verschiedene Weisen und kostenlos erreicht werden. Beispielhafte Möglichkeiten hierfür wären ein Google-My-Business-Eintrag oder die Präsenz in den sozialen Medien. Durch die Identifikation der Netzwerke als bedeutender Informationsvermittler ist es für die Inhaber ratsam, in einem solchen zu agieren, um so von dem Erfahrungsaustausch, den Hilfestellungen und leicht zugänglichen Informationen zu profitieren. Besonders in Krisensituationen wie der Coronapandemie hat sich die Teilnahme in einem Netzwerk als hilfreich und unterstützend herausgestellt. Des Weiteren wird von den Experten betont, dass gut vernetzte Inhaber besser durch die Pandemie gekommen sind als auf sich alleine gestellte.

Für den Einstieg in den Onlinehandel wurden verschiedene Ansätze von den Experten empfohlen. Grundsätzlich sollte eine Abwägung bei der Auswahl der passenden digitalen KKM stattfinden. An dieser Stelle spiegeln die Branche sowie die Zielgruppe bedeutsame Komponenten wider. Für eine schnelle Präsenz und Verkauf eignet sich der LOM, welcher eine beliebte Alternative zu den großen OM (z. B. Amazon oder eBay) darstellt. Für eine langfristige Kundenbindung bietet der eigene Online-Shop Vorteile. Dieser setzt allerdings mehr Kenntnisse voraus und nimmt mehr Zeit in Anspruch.

Gleichwohl lassen die Aussagen der Inhaber aber erahnen, dass insbesondere die sozialen Medien aktuell und zukünftig von großer Bedeutung sind. Die bereits in der Diskussion aufgeworfene Frage, ob *new social commerce* zukünftig einen eigenen Online-Shop für Inhaber überflüssig macht, bringt eine weiterführende Forschungsfrage mit sich. Letztendlich stellt der Beitrag heraus, dass die Digitalisierung der Kundenkommunikation eine notwendige und unausweichliche Investition für die Zukunft ist.
